# Extracellular vesicle proteomics in staphylococcus aureus-treated blood and sepsis reveals coordinated complement, acute phase, neutrophil, and exocytosis responses

**DOI:** 10.1038/s41598-026-54598-7

**Published:** 2026-05-30

**Authors:** Dapi Menglin Chiang, Michael W. Pfaffl, Gustav Schelling, Agnes S. Meidert, Florian Brandes, Christina Ludwig, Susanne I. Wudy, Benedikt Kirchner, Mia S. C. Yu, Christian Zenner, Rosalie Ulbricht, Laurent Muller, Marlene Reithmair

**Affiliations:** 1 Division of Animal Physiology and Immunology, School of Life Sciences Weihenstephan, Technical University of Munich (TUM), 85354 Freising, Germany; 2 Institute of Human Genetics, University Hospital, LMU Munich, 80336 Munich, Germany; 3 Department of Anaesthesiology, LMU University Hospital Munich, 81377 Munich, Germany; 4 Bavarian Center for Biomolecular Mass Spectrometry (BayBioMS), TUM School of Life Sciences, Technical University of Munich, Freising, Germany; 5 Intestinal Microbiome, ZIEL - Institute for Food & Health, School of Life Sciences, Technical University of Munich (TUM), Freising, Germany; 6 Department of Otorhinolaryngology, Head and Neck Surgery, University Hospital of Basel, Basel, Switzerland; 7 Department of Biomedicine, University of Basel, Basel, Switzerland

**Keywords:** Diseases, Immunology, Microbiology

## Abstract

Sepsis accounts for nearly 20% of global mortality, with antibiotic resistance worsening clinical outcomes. Rapid antibiotic administration and accurate pathogen identification remain crucial. It is well now known that extracellular vesicles (EVs) from human cells and bacterial membrane vesicles (bMVs) play a central role in the interaction between host and pathogen and represent promising biomarkers for early infections. This study investigated how antibiotic exposure alters EV responses in *Staphylococcus aureus* (SA)spiked blood and compared these findings with EV proteome profiles from bacteremia patients. In an in vitro model, whole blood from healthy donors was spiked with SA at a multiplicity of infection (MOI) of 0.001, treated with clinically relevant concentrations of piperacillin–tazobactam, vancomycin, or moxifloxacin, and plasma was subsequently isolated for EV analysis. EVs were isolated using the Miltenyi Pan EV Kit and analyzed by bead-based flow cytometry and high-resolution LC–MS/MS. In parallel, serum EVs from healthy controls (*n* = 6) and bacteremia patients (*n* = 12; 6 blood culture–positive and 6 culture-negative) were analyzed using the same workflow. Flow cytometry revealed increased levels of CMO⁺ CD45⁺ PanEV⁺ SA⁺ vesicles in SA-spiked samples, particularly following low-dose piperacillin–tazobactam and high-dose vancomycin treatment, despite minimal changes in vesicle size and total particle counts. Proteomic analysis of plasma EVs showed significant alterations in protein composition, including increased abundance of the SA-derived ribosomal protein rplU and host defense–associated proteins. Functional enrichment highlighted pathways related to neutrophil degranulation, vesicle-mediated transport, and antibacterial responses. In patient samples, serum EVs were enriched in acute-phase and immune-related proteins, including SERPINA1, SERPINA3, CRP, and SAA2, along with canonical EV markers such as CD81 and syntenin-1, irrespective of blood culture status. Antibiotic exposure and SA infection are associated with measurable changes in the human EV proteome, characterized by enrichment of immune and host defense–related proteins despite stable vesicle numbers. Similar EV-associated protein patterns were observed in both blood culture–positive and –negative patient samples, reflecting shared features of the systemic host response to infection and highlighting the potential of EV profiling to capture infection-associated biological signals.

**Supplementary Information:**

The online version contains supplementary material available at 10.1038/s41598-026-54598-7.

## Introduction

Sepsis is a severe, life-threatening syndrome in which the host’s response to infection becomes dysregulated, triggering systemic inflammation, tissue injury, and the risk of multiple organ failure^1^. Sepsis continues to affect millions worldwide, with about 49 million cases and 11 million deaths each year, and the burden falls most heavily on low- and middle-income nations^1^. Sepsis commonly develops when bacteria from a localized infection enter the bloodstream. In community-onset sepsis, frequent bloodstream pathogens include *Escherichia coli*, *Klebsiella* species, *Streptococcus* species, and *Enterococcus* species^2^. *Staphylococcus aureus* (SA), including both methicillin-susceptible (MS) SA and methicillin-resistant (MR) SA strains, respectively MSSA and MRSA, is often found in blood cultures, and MRSA infections are associated with higher hospital mortality^2^. In sepsis involving SA, immune homeostasis becomes severely disrupted, with strong inflammatory activation occurring alongside an immunosuppressive state that allows the bacteria to persist and spread^3,4^.

In early sepsis, some well-known protein biomarker like S100A8/A9, pentraxin-3 (PTX3), serum amyloid A1 (SAA1), high mobility group box 1 (HMGB1), resistin, and complement C3/C5 are elevated and reflect disease severity. These key host responses, including neutrophil, endothelial, and complement activation, are reflected in proteins such as SAA1 and PTX3; however, their detection remains challenging due to low baseline levels, rapid fluctuations during illness, and interference from abundant plasma proteins^3,5–7^. In contrast, clinically used markers such as C-reactive protein (CRP) are nonspecific and can give a false sense of reassurance, since CRP may remain low even in confirmed sepsis, particularly in the early phase of illness or in immunocompromised patients, which can delay timely diagnosis and treatment^8^. Together, these issues reveal the constraints of relying solely on untargeted plasma proteomics and suggest that extracellular vesicles (EVs) may provide a richer and more mechanistically relevant pool of biomarkers.

EVs released by human cells, particularly small EVs (sEVs, 30–150 nm) formerly known as exosomes, carry cargo that reflects their cellular origin, and unlike low-abundance or rapidly cleared plasma proteins, they provide a concentrated and protected source of mechanistically informative mediators, making them valuable players and biomarker candidates in sepsis^9,10^. Bacteria also secrete vesicles of similar size, known as bacterial membrane vesicles (bMVs), which carry virulence factors and immunomodulatory molecules that can contribute to sepsis pathogenesis^11^. In sepsis, sEVs are actively released by immune cells, endothelial cells, epithelial cells, and platelets in response to infection, hypoxia, and systemic inflammation, contributing to both the propagation and regulation of host immune responses^10,12^. Proteome profiles of sEVs in sepsis patients often show higher levels of inflammation-related proteins, including SAA1, CRP, and immunoglobulin components, suggesting that these vesicles reflect key inflammatory processes and may aid in diagnosis^13^. Monocyte- and platelet-derived EVs in sepsis carry surface markers and cargo that promote endothelial activation, increase vascular permeability, and enhance tissue factor–mediated coagulation, directly contributing to microvascular dysfunction and multi-organ failure^14^. Although interest in sEVs as biomarkers in sepsis is growing, the dynamic response of the host sEVs proteome to direct bacterial challenges, such as SA infection, remains poorly understood. Furthermore, how antibiotic treatment shapes this response remains unclear because comprehensive comparisons among culture-positive sepsis, culture-negative sepsis, and healthy individuals are limited, even though such analyses are essential for defining diagnostic specificity and clinical usefulness. In this study, we investigated the human EV proteomic landscape using a dual approach: (1) an in vitro whole-blood infection model challenged with SA, with or without antibiotic treatment, and (2) clinical plasma samples from patients with blood culture–positive and –negative sepsis, alongside healthy volunteers. Using immunomagnetic bead–based EV isolation together with label-free proteomics and multivariate analysis, we set out to define EV signatures that track with infection status and treatment effects. This approach allowed us to detect both human and bacterial proteins within the vesicles and to observe how antibiotic exposure influenced their composition. Building on these findings, we compared EV proteomes from culture-positive sepsis, culture-negative sepsis, and healthy individuals to define EV signatures that reflect infection status and treatment response in sepsis.

## Methods

### SA-spiked plasma sample collection

Peripheral blood (55 ml) was collected from six healthy volunteers in EDTA-coated tubes to prevent coagulation. From each donor, 5 ml of whole blood was distributed into 15 ml Falcon tubes and allocated to 11 experimental conditions: a MOCK (non-infection) control, infection with SA DSM 20,231 (MSSA), and infection in combination with nine antibiotic treatments. The SA DSM 20,231 strain was obtained from DSMZ (Deutsche Sammlung von Mikroorganismen und Zellkulturen, German Collection of Microorganisms and Cell Cultures, Braunschweig, Germany) and maintained on tryptic soy broth (TSB) agar plates. A single colony was inoculated into 500 ml of RPMI-1640 medium, prepared as two 250-ml cultures, and grown at 37 °C, yielding a bacterial suspension with an OD₆₀₀ of approximately 1.0–1.5. From the resulting culture mixture, a 1-ml aliquot was labeled with the CFSE Cell Division Tracker Kit (BioLegend, San Diego, USA) for 30 min at 37 °C. CFSE-labeled SA were quantified by flow cytometry (LSRFortessa™, Becton, Dickinson and Company, Franklin Lakes, NJ, USA), and data analysis was performed using FlowJo 10.8 (Becton, Dickinson and Company, Franklin Lakes, NJ, USA).

For infection, 3 × 10⁷ SA were added to 5 mL of whole blood, corresponding to a bacterial concentration of 6 × 10⁶ cells/mL. The whole blood cell count was approximately 6 × 10⁹ cells/mL (i.e., 3 × 10¹⁰ cells in 5 mL), resulting in a multiplicity of infection (MOI) of 0.001 (bacteria-to-host cell ratio), as determined using a TC10™ Automated Cell Counter (Bio-Rad Laboratories, CA, USA). Antibiotic concentrations were selected to represent clinically relevant plasma levels: piperacillin–tazobactam (25, 50, 100 µg/ml), vancomycin (6.25, 12.5, 25 µg/ml), and moxifloxacin (0.18125, 0.725, 2.9 µg/ml). These antibiotics were selected for their distinct mechanisms of action, including inhibition of cell wall synthesis by piperacillin–tazobactam (Pip-Tazo) and vancomycin, and inhibition of DNA replication by moxifloxacin, in accordance with the Surviving Sepsis Campaign 2021 international guidelines for empiric treatment of SA and sepsis^15^. All 11 experimental conditions were used for flow cytometric analysis, whereas only the highest concentration of each of the three antibiotics (100 µg/ml Pip-Tazo, 25 µg/ml vancomycin, and 2.9 µg/ml moxifloxacin), together with the MOCK control and infection-alone condition, was used for subsequent proteomic analyses. Following 24 h incubation at 37 °C, plasma was separated by centrifugation at 2,000 × g for 10 min at 4 °C and filtered through a 0.45 μm PES syringe filter (Merck KGaA, Darmstadt, Germany) to remove residual bacteria. Approximately 2 ml of filtered plasma was collected per condition and stored at − 80 °C until analysis.

### Human EV isolation

Human EVs were isolated from (i) 1 ml of filtered plasma obtained from the SA-spiked blood model (MOCK control, infection-only, and antibiotic-treated conditions; *n* = 6 per condition) and (ii) serum EV–Miltenyi bead complexes collected from healthy controls (HC, *n* = 6), bacterial culture–negative patients (BCN, *n* = 6), and bacterial culture–positive patients (BCP, *n* = 6). EV isolation was performed using immunoaffinity capture with the EV Isolation Kit Pan, human (Miltenyi Biotec, Bergisch Gladbach, Germany), following the manufacturer’s protocol. Briefly, 1 ml of filtered plasma or serum was incubated with 50 µl of immunoaffinity beads for 1 h on an overhead rotor. A Miltenyi µColumn was equilibrated and washed three times before sample loading, followed by four additional washes on a magnetic stand. EV–Miltenyi bead complexes were eluted using 100 µl of the kit-supplied isolation buffer. The unbound plasma fraction (1 ml) collected during the immunoaffinity procedure was retained for additional downstream analyses. Clinical characteristics of all patients, including age, sex, hospital and intensive care stay, pathogen distribution, day 1 antibiotic treatment status, and day 1 inflammatory markers (CRP, interleukin-6, leukocyte count, and procalcitonin), are summarized in Table 1.

**Table 1:** Summary of clinical characteristics.

Characteristics	BCP (*n* = 6)	BCN (*n* = 6)
Age (years) – Mean ± SD	67.8 ± 16.7	74.8 ± 15.7
Male	4	3
Female	2	3
Hospital stay – Mean ± SD	3.8 ± 2.8	9.3 ± 6.8
Intensive care – Mean ± SD	3.0 ± 2.7	3.7 ± 3.2
Pathogens	SA (4), *S. capitis* (1), *C. tertium* (1)	None
Day 1 antibiotic treatment – Yes	5	3
Day 1 antibiotic treatment – No	1	3
Day 1 C-reactive protein	22.7 ± 16.3	22.4 ± 14.5
Day 1 Interleukin-6	567.9 ± 590.2	199.8 ± 203.5
Day 1 Leukocytes count	11.1 ± 7.1	20.2 ± 13.8
Day 1 Procalcitonin	13.9 ± 12.4	2.9 ± 3.1

### In-house bMV isolation

The bacterial beads (BacB) necessary for the bMVs isolation is described in brief: First, 250 µl of 40 µg/ml 1-ethyl-3-(3-dimethylaminopropyl) carbodiimide hydrochloride (EDC) (Thermo Fisher Scientific, Bremen, Germany) in 0.1 M 2-(N-morpholino)ethanesulfonic acid (MES) buffer (Sigma + Merck KGaA, Darmstadt, Germany), pH 5.0, was used to activate 10 mg of carboxyl-group magnetic particles (2.13 × 10¹⁰ particles/ml, IKERLAT Polymers, Lasarte-Oria, Spain) at 25 °C for 15 min. The activated magnetic particles were then resuspended in 1 ml of PBS. Next, 50 µg each of OmpA antibodies (catalog number: orb26813, Biorbyt, Cambridge, UK) and GroEL antibodies (catalog number: NBP2-32867, Novus Biologicals, Centennial, United States) were prepared. A total of 100 µl of the antibody mixture was incubated with 1 ml of activated magnetic particles at 37 °C for 2 h. To block unoccupied binding sites, the antibody-coated beads were incubated with 1 ml of 10% EV-free BSA in PBS (prepared by centrifugation at 100,000 × g, 4 °C, overnight) at 37 °C for 30 min. The resulting beads were then diluted to 1 × 10⁶ beads/µl and used as BacB. One milliliter of unbound plasma was incubated with 100 µl of BacB at 37 °C for 1 h. After incubation, bMV–BacB complexes were washed twice with 1 ml of 0.5% EV-free BSA in PBS while magnetic bMV–BacB complexes were retained in the tube using a magnetic stand. As a negative control, 1 ml of 10% EV-free (EF) BSA in PBS was incubated with BacB under the same conditions to generate EF–BacB complexes. bMV–BacB complexes were used for further analysis by bead-based flow cytometry or proteomics, while EF–BacB complexes were used only as a negative control for bead-based flow cytometry.

### Bead-based flow cytometry

25 µl of EV–Miltenyi bead complexes or 250 µl of bMV–BacB complexes were diluted in 0.9 ml of 0.5% EV-free BSA in PBS. To this mixture, 100 µl of an antibody master mix was added. The master mix contained 2.5 µl of PanEV antibody (3 µg), pre-mixed with 1 µl each of PE-conjugated anti-CD63 (clone H5C6), PE-conjugated anti-CD81 (clone 5A6), and PE-conjugated anti-CD9 (clone HI9a) (all from BioLegend, San Diego, USA); 5 µl of Brilliant Violet 421™ anti-human CD45 antibody (clone HI30, BioLegend, San Diego, USA); 10 µM CellMask™ Plasma Membrane Deep Red (CMD; C10046, Thermo Fisher Scientific, Bremen, Germany); and 1 µl of FITC-conjugated *SA* polyclonal antibody (PA1-73172, Thermo Fisher Scientific, Bremen, Germany). The mixture was incubated at 37 °C for 1 h. After staining, EV–Miltenyi bead complexes and bMV–BacB complexes were washed twice with 1 ml of 0.5% EV-free BSA in PBS and centrifuged at 100,000 × g at 4 °C overnight using a magnetic rack. The samples were then analyzed on a BD LSR Fortessa™ flow cytometer (Becton, Dickinson and Company, Franklin Lakes, USA). Data analysis, including gating, was performed using FlowJo software version 10.8 (Becton, Dickinson and Company, Franklin Lakes, USA). An IgG isotype control was included to confirm the specificity of the staining. In the flow cytometry gating strategy, EV–Miltenyi bead complexes and bMV–BacB complexes were initially gated based on forward scatter (FSC-A) and side scatter (SSC-A). The CMD⁺ PanEV⁺ CD45⁺ Anti-SA⁺ population from the EV–Miltenyi bead complexes was identified as immune cell-derived EVs carrying SA antigen. In contrast, the CMD⁺ PanEV⁻ Anti-SA⁺ population from the bMV–BacB complexes was identified based on comparison to the isotype control.

### Human EV characterization

Fluorescence nanoparticle tracking analysis (NTA) was used to determine the size distribution and particle concentration of extracellular vesicles (EVs) using ZetaView^®^ PMX 110 or x30 MONO instruments equipped with a 520-nm laser and a 550-nm emission filter (Particle Metrix GmbH, Inning am Ammersee, Germany). EV samples were diluted in particle-free PBS to fall within the optimal measurement range of the instrument and were measured in two acquisition cycles across 11 positions per sample. For fluorescence measurements, EV membranes were labeled with CellMask Orange™ (CMO; 5 mg/mL stock; Thermo Fisher Scientific Inc., Waltham, MA, USA) at a final concentration of 25 µg/mL (1:200 dilution), and samples were further diluted 1:400 prior to analysis unless otherwise stated. Fluorescence measurements on the PMX 110 system were performed using 1 mL of filtered plasma obtained from a *Staphylococcus aureus*–spiked blood model (mock control, infection-only, and antibiotic-treated conditions; *n* = 6 per group) with video capture settings of shutter speed 70, sensitivity 95, and a frame rate of 30 frames/s, and data were analyzed using ZetaView software version 8.05.11 SP1. Fluorescence measurements on the x30 MONO system were conducted on serum EV–Miltenyi bead complexes from healthy controls (HC; *n* = 6), bacterial culture-negative patients (BCN; *n* = 6), and bacterial culture-positive patients (BCP; *n* = 6) using a shutter speed of 100, sensitivity 90, and a frame rate of 30 frames/s, with data analyzed using ZetaView software version 8.06.01 SP1 (Particle Metrix GmbH, Inning am Ammersee, Germany).

Cryo-electron microscopy (cryo-EM) sample preparation was conducted as previously described^16^. In brief, 4 µl of each sample was applied to glow-discharged holey carbon-coated grids (Ted Pella Inc., Redding, United States), blotted using Whatman No. 1 filter paper, and vitrified by plunge-freezing into liquid ethane at − 178 °C with a Leica GP plunger (Leica Microsystems GmbH, Wetzlar, Germany). Vitrified grids were transferred to a Talos electron microscope (FEI/Thermo Fisher Scientific Inc., Waltham, United States) using a Gatan 626 cryo-holder (Gatan Inc., Pleasanton, United States). Imaging was performed at an accelerating voltage of 200 kV under low-dose conditions (20 e⁻/Å²) while maintaining cryogenic temperatures. Micrographs were acquired using a CETA camera (Thermo Fisher Scientific Inc., Waltham, United States).

Negative staining transmission electron microscopy (TEM) was performed as previously described^16^. Briefly, 5 µl of undiluted sample was adsorbed for 60 s onto glow-discharged parlodion/carbon-coated copper grids (FCF200-Ni; Electron Microscopy Sciences, Hatfield, United States). The grids were then blotted, rinsed three times with double-distilled water (ddH₂O), and stained on two consecutive droplets of 2% uranyl acetate solution (Merck KGaA, Darmstadt, Germany). Imaging was carried out using a Talos F200C TEM (FEI/Thermo Fisher Scientific Inc., Waltham, United States) operated at an accelerating voltage of 120 kV. Electron micrographs were acquired using a Veleta camera (EMSIS GmbH, Münster, Germany).

### Protein preparation for proteomics

EV proteins from (i) plasma EV–Miltenyi bead complexes, unbound plasma bMV–BacB complexes, and total plasma obtained from the SA-spiked blood model (MOCK control, infection-only, and antibiotic-treated conditions; *n* = 6 per condition), and from (ii) serum EV–Miltenyi bead complexes collected from healthy controls (HC, *n* = 6), bacterial culture–negative (BCN, *n* = 6), and bacterial culture–positive (BCP, *n* = 6) patient samples were lysed in 1× RIPA buffer (Abcam plc, Cambridge, United Kingdom; ab156034) supplemented with 1× ProteaseArrest™ Protease Inhibitor Cocktail (G-BIOSCIENCES, St. Louis, MO, United States), and stored at − 80 °C until further use. Protein samples were then boiled at 70 °C for 10 min and sonicated on ice for 5 min. Following sonication, lysates were centrifuged at 10,000 × g at 4 °C for 30 min. Protein concentrations were determined using the bicinchoninic acid (BCA) assay (Thermo Fisher Scientific Inc., Waltham, United States). A minimum of 5 µg of protein from each sample was mixed with 1× Laemmli Sample Buffer (Bio-Rad Laboratories, Hercules, United States) containing 2-mercaptoethanol (Merck KGaA, Darmstadt, Germany) in a final volume of 35 µl. Prepared samples were then submitted to the proteomics core facility at the Bavarian Center for Biomolecular Mass Spectrometry for analysis.

### Mass spectrometry-based proteomics

In accordance with standard procedures, in-gel trypsin digestion was performed^17^ on all samples derived from both experimental datasets. This included (i) EV–Miltenyi bead complexes, bMV–BacB complexes, and total plasma obtained from the SA-spiked blood model (MOCK control, infection-only, and antibiotic-treated conditions; *n* = 6 per condition), as well as (ii) EVs and total plasma or serum collected from healthy controls (HC, *n* = 6), bacterial culture–negative (BCN, *n* = 6), and bacterial culture–positive (BCP, *n* = 6) patient samples. Briefly, each sample was loaded onto a NuPAGE™ 4–12% Bis-Tris protein gel (Thermo Fisher Scientific, Waltham, USA) and run approximately 1 cm into the gel to concentrate the proteins into a single non–size-separated band. The accumulated band was excised, reduced with 50 mM dithiothreitol, alkylated with 55 mM chloroacetamide, and digested overnight at 37 °C with Trypsin Gold (mass spectrometry grade, Promega). Following digestion, peptides were extracted, dried, and resuspended in 25 µl of buffer A (2% acetonitrile, 0.1% formic acid in HPLC-grade water). A 5 µl aliquot of each peptide solution was injected for LC–MS/MS analysis.

LC-MS/MS measurements were conducted using a Dionex Ultimate 3000 RSLCnano system linked to an Orbitrap Fusion LUMOS instrument (ThermoFisher Scientific, Bremen), in adherence to the standard protocol of the core facility^18,19^. Samples were initially loaded onto a self-packed trap column (ReproSil-pur C18-AQ, 5 μm, 20 mm × 75 μm, Dr. Maisch) at a flow rate of 5 µL/min using 0.1% formic acid. After a 10-minute loading step, peptides were eluted onto a self-packed analytical column (ReproSil Gold C18-AQ, 3 μm, 450 mm × 75 μm, Dr. Maisch). Chromatographic separation occurred over a 50-minute gradient increasing from 4% to 32% solvent B (acetonitrile containing 0.1% FA and 5% DMSO) against solvent A (water containing 0.1% FA and 5% DMSO) at a flow rate of 300 nL/min. The mass spectrometer operated in positive mode using data-dependent acquisition (DDA). We acquired full MS1 scans (360–1300 m/z) at a resolution of 60,000, with a normalized AGC target of 100% and a maximum injection time of 50 ms. A 2-second cycle time was employed, selecting precursors with charge states between 2 and 6, while applying a 30-second dynamic exclusion. Fragmentation was carried out via higher-energy collision-induced dissociation (HCD) at 30% normalized collision energy (NCE) with a 1.3 m/z isolation window. MS2 spectra were recorded at a resolution of 15,000, using an AGC target of 150% and a maximum injection time of 22 ms.

### Label-free quantitative proteomics and integrated statistical analysis for host–pathogen profiling in sepsis

Proteomic peptide identification and quantification were executed in accordance with our previous study^19^, utilizing the MaxQuant computational platform (version 1.6.3.4) and the integrated Andromeda search algorithm^20^. Tandem mass spectrometry (MS/MS) spectra were queried against UniProt reference proteomes for *Homo sapiens* (Taxon ID: 9606, UP000005640, July 2020) and *Staphylococcus aureus* (Taxon ID: 1280, July 2024), with the search space augmented by the standard MaxQuant contaminant database. Search parameters were configured with Trypsin/P specificity; carbamidomethylation of cysteine was defined as a fixed modification, while methionine oxidation and N-terminal acetylation were treated as variable modifications. To ensure statistical reliability, a target-decoy strategy utilizing reversed sequences was applied, restricting the false discovery rate (FDR) to 1% at both the peptide spectrum match (PSM) and protein levels. Subsequent data curation was conducted in Perseus (version v2.0.10.0)^21^. The dataset was filtered to exclude proteins identified merely by site, reverse hits, and potential contaminants, after which label-free quantification (LFQ) intensities underwent log10 transformation^21^.

To identify proteins reproducibly detected across sample types including EV–Miltenyi bead complexes, bMV–BacB complexes, total plasma, and patient serum, proteins were retained if they had valid values in at least 50% of biological replicates within any one group, using the “Filter rows based on valid values” function in Perseus^21^. Missing values were imputed using the standard normal distribution–based imputation in Perseus, followed by quantile normalization using the built-in Perseus normalization function. These preprocessing steps were applied consistently to both datasets: (i) the *SA*–spiked plasma experiment and (ii) the patient cohort comprising HC, BCN, and BCP groups.

Preprocessed data were subsequently analyzed in RStudio (version: 2025.05.0 + 496)^22^ with R (version: 4.5.1)^23^. Differential expression analysis was performed separately for the two datasets. For the SA–spiked blood experiment (i), paired two-sided t-tests were applied to account for matched biological replicates from the same donors across conditions, with p-values adjusted using the Benjamini–Hochberg (BH) procedure and reported as adjusted p-values (FDR). For the patient serum cohort (ii), unpaired two-sided Welch’s t-tests were used to account for unequal variance between independent clinical samples, with BH-adjusted p-values reported. In both analyses, proteins were considered significantly regulated if they exhibited an adjusted p-value < 0.05 and an absolute log₁₀ fold change (|log₁₀FC|) greater than 0.301 (corresponding to a fold change > 2 or < 0.5). Volcano plots were generated using ggplot2, and significant proteins were annotated using a combined human and *Staphylococcus aureus* protein database. Functional enrichment analysis was performed using clusterProfiler (version: 4.14.6)^24–27^, ReactomePA (version: 1.50.0)^28^, and org.Hs.eg.db (version: 3.20.0) to identify enriched Gene Ontology (GO), KEGG^29^, and Reactome pathways, with significance assessed using Fisher’s exact test and results visualized via pathway–protein heatmaps filtered by sepsis-related keywords. To assess global expression patterns, sparse Partial Least Squares Discriminant Analysis (sPLS-DA) was performed using 2 (version 6.30.0)^30^, with non-finite and zero-variance features removed prior to analysis; model tuning was conducted using repeated 5-fold cross-validation (10 repeats) optimizing keepX (25, 50, 100) based on Balanced Error Rate (BER) and centroid distance, and performance was evaluated using repeated M-fold cross-validation and confusion matrices. Given the modest sample size, sPLS-DA was used as an exploratory approach, and group separation was interpreted cautiously. Component plots with confidence ellipses were generated in RStudio (version 2025.05.0 + 496)^22^ with R (version 4.5.1)^23^, and Venn diagrams were constructed using the VennDiagram package to visualize overlap of differentially expressed proteins.

## Results

**Fig. 1:**
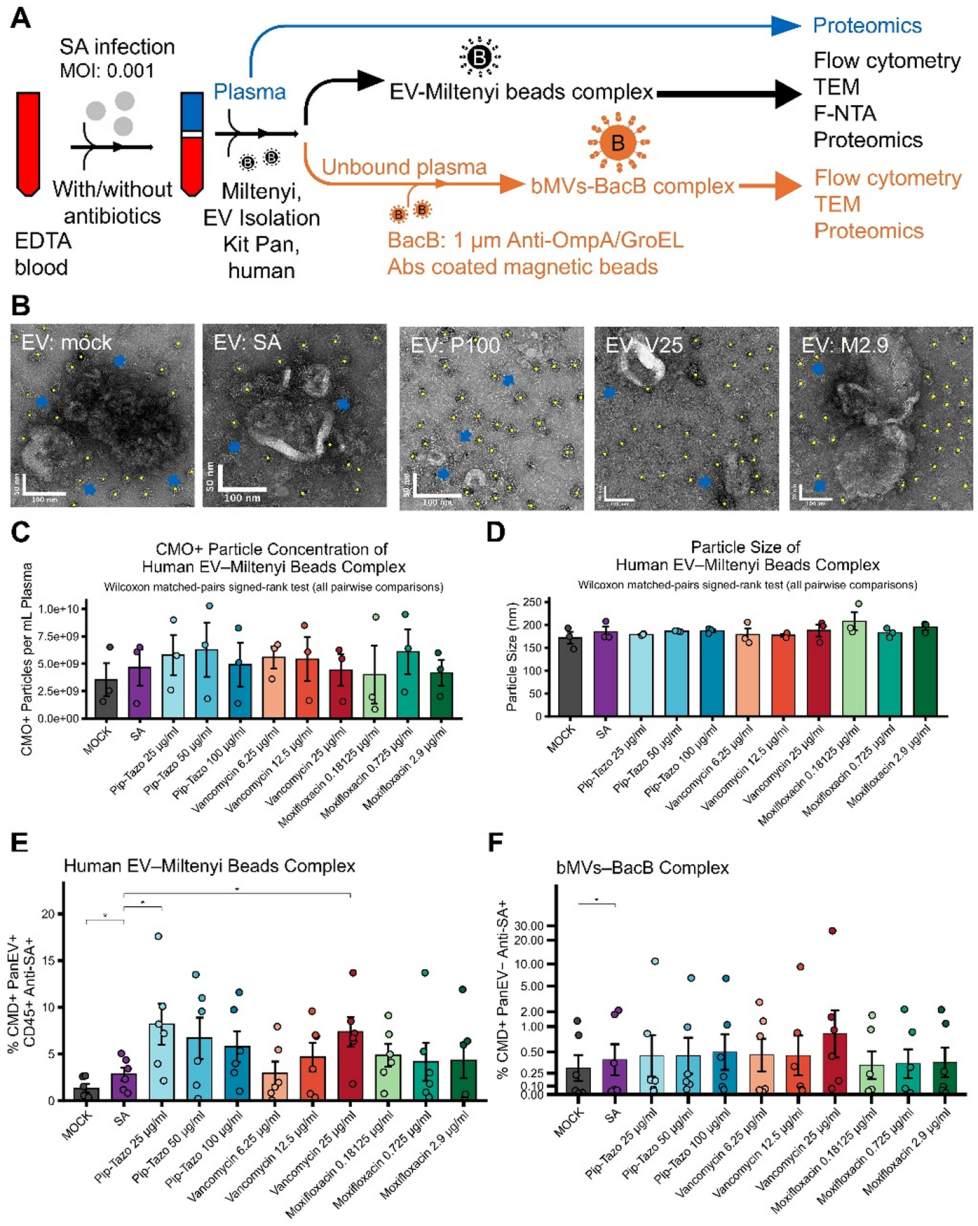
Workflow and analysis of plasma EVs and bMVs during *S. aureus* (SA) infection with or without antibiotic treatment. (**A**) EVs and bMVs were isolated from EDTA plasma using Miltenyi and BacB beads, respectively, and analyzed by flow cytometry, transmission electron microscopy (TEM), fluorescence nanoparticle tracking analysis (F-NTA), and proteomics. (**B**) Representative TEM images show EV–Miltenyi bead complexes under different treatment conditions, with yellow dots indicating Miltenyi beads and blue arrows indicating vesicles (P100 = piperacillin–tazobactam 100 µg/mL, V25 = vancomycin 25 µg/mL, M2.9 = moxifloxacin 2.9 µg/mL; scale bars, 100 nm). (**C–D**) Bar plots with overlaid scatter points show mean ± SEM of F-NTA particle size (**C**) and CMO⁺ particle concentration (**D**) of EV–Miltenyi bead complexes across treatment groups; individual dots represent paired biological replicates (*n* = 3). Statistical significance was assessed using paired Wilcoxon matched-pairs signed-rank tests for all pairwise comparisons; piperacillin–tazobactam is abbreviated as Pip-Tazo. (**E–F**) Bar plots with overlaid scatter points show mean ± SEM percentages of (E) CMD⁺ PanEV⁺ CD45⁺ anti-SA⁺ EV–Miltenyi bead complexes and (**F**) CMD⁺ PanEV⁻ anti-SA⁺ bMV–BacB bead complexes quantified by flow cytometry across treatment groups; individual dots represent paired biological replicates (*n* = 6). Statistical significance was assessed using paired Wilcoxon matched-pairs signed-rank tests.

To assess the impact of SA infection, we isolated EVs and bMVs from plasma using two approaches: Miltenyi beads for human EV capture and BacB-coated magnetic beads for bMV–EV complex enrichment (Fig. 1A). F-NTA and negative-stain electron microscopy of the EV–Miltenyi bead complexes first showed no observable differences across all experimental groups (Fig. 1B–D). Negative-stain electron microscopy of bMV-BacB complexes also showed no observable differences across all the groups (Supplemental Fig. 1A-E). Analysis of the EV–Miltenyi bead complexes revealed a significant increase in CMD⁺ PanEV⁺ CD45⁺ *SA*^*+*^ signals in the SA-spiked group compared to the MOCK control (*p* = 0.032) (Fig. 1E). Within the SA-spiked groups, treatment with either the lowest concentration of piperacillin-tazobactam (25 µg/ml) (*p* = 0.032) or the highest concentration of vancomycin (25 µg/ml) (*p* = 0.032) also resulted in significantly higher CMD⁺ PanEV⁺ CD45⁺ SA⁺ signals compared to the SA-spiked group without antibiotics (Fig. 1E). Analysis of the bMV fraction, using unbound plasma collected after Miltenyi bead isolation, revealed that the bMV–BacB complexes exhibited a significant increase in CMD⁺ PanEV^−^ SA⁺ signals in the SA-spiked group compared to the MOCK control (*p* = 0.032) (Fig. 1F). However, no significant differences were observed between the antibiotic-treated groups and the SA-spiked group without treatment (Fig. 1F). These findings suggest that SA infection induces significant changes in the protein composition of EVs and bMVs, rather than altering their total quantity, as indicated by EV flow cytometry and F-NTA.

**Fig. 2:**
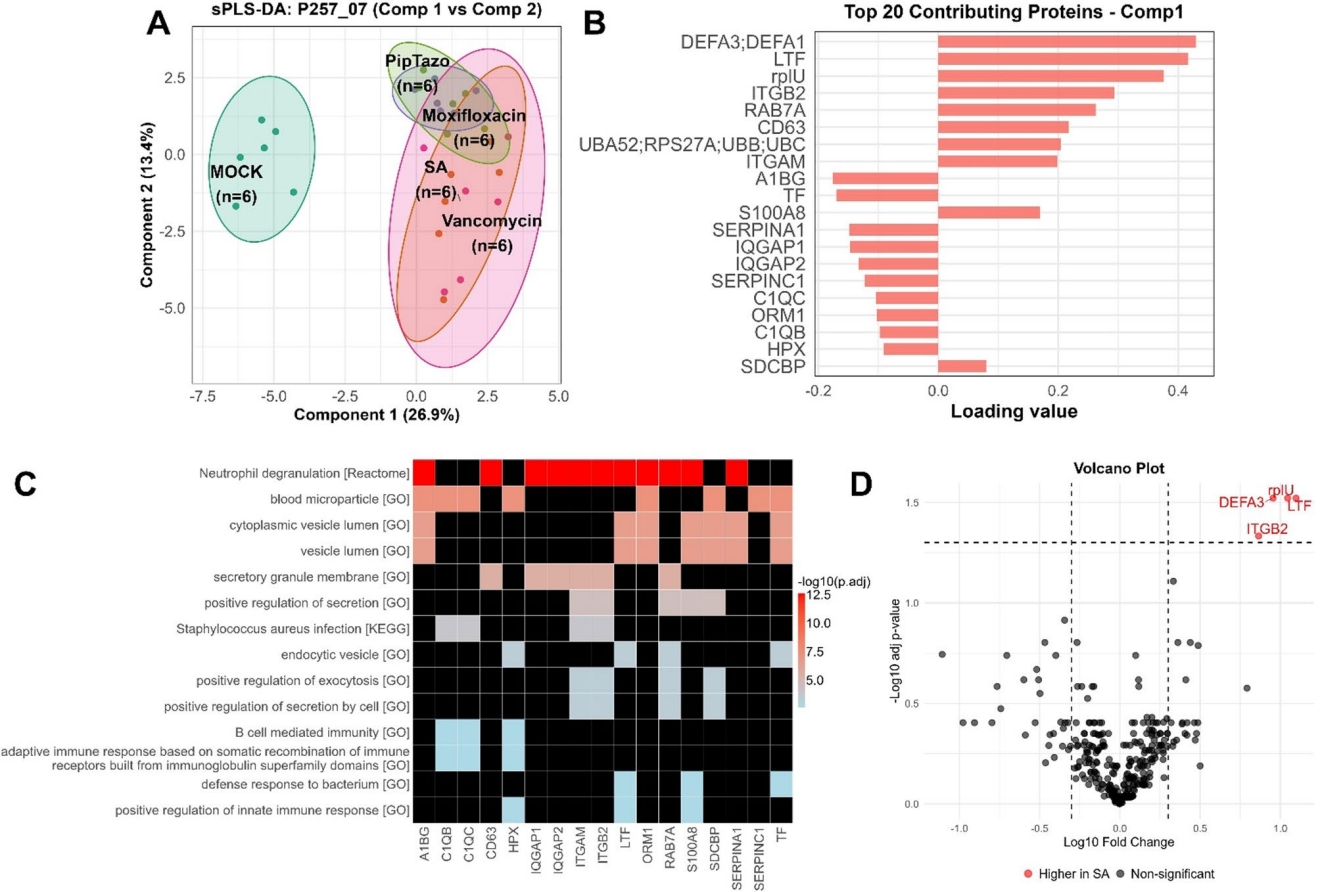
Proteomic profiling of plasma EVs reveals SA infection- and antibiotic-specific signatures. (**A**) sPLS-DA plot (components 1 vs. 2) showing distinct clustering of EV proteomes from MOCK, SA-infected, 100 µg/mL piperacillin–tazobactam (PipTazo), 25 µg/mL Vancomycin, and 2.9 µg/mL Moxifloxacin groups (*n* = 6 per group). Ellipses represent 95% confidence intervals. (**B**) Top 20 proteins contributing to component 1 separation, with highest loading values from SA-infected samples. (**C**) Pathway enrichment analysis of the top 20 component 1 proteins, showing enrichment in secretion, vesicle trafficking, and immune response pathways. Significance was assessed using Fisher’s exact test with Benjamini–Hochberg (BH) correction; color scale represents −log10(adjusted p-value). (**D**) Volcano plot comparing SA vs. MOCK groups, highlighting significantly upregulated (red) and downregulated (blue) proteins. Significance was determined using pairwise *t*-tests with BH correction, based on adjusted p-value and log10 fold change thresholds.

To better understand how SA infection, with or without antibiotic treatment, alters the protein composition of EVs and bMVs, we performed proteomic profiling of both fractions using antibiotic concentrations that produced the strongest effects in our flow cytometry assays, with emphasis on the highest concentrations to approximate clinically achieved plasma levels and ensure physiologically relevant exposure^15,31–33^, alongside total plasma proteomics to capture systemic protein changes. However, protein yields from the bMV fraction and total plasma were insufficient for reliable pairwise statistical comparisons (Supplementary Fig. 2A–B), and subsequent analyses were therefore focused on the human EV fraction. sPLS-DA analysis revealed separation between MOCK controls and SA-infected or antibiotic-treated groups (Fig. 2A), with model tuning performed using repeated M-fold cross-validation and balanced error rate (BER) minimization to evaluate performance across components. The top contributors to component 1 included DEFA3, LTF, the *Staphylococcus aureus*–derived ribosomal protein rplU, ITGB2, RAB7A, CD63, UBA52, ITGAM, A1BG, TF, S100A8, SERPINA1, IQGAP1, IQGAP2, SERPINC1, C1QC, ORM1, C1QB, HPX, and SDCBP (Fig. 2B). Functional enrichment analysis of these proteins showed significant associations with neutrophil degranulation, vesicle-mediated transport, SA infection, defense response to bacterium, and immune-related pathways (adjusted p-value < 0.05; Fig. 2C). Univariate differential analysis using paired t-tests with Benjamini–Hochberg correction identified DEFA3, LTF, RplU, and ITGB2 as significantly increased in the SA infection group relative to MOCK controls, as visualized by a volcano plot (adjusted p-value < 0.05 and fold change > 2; Fig. 2D), with complementary analyses based on unadjusted p-values shown in Supplementary Fig. 2C–F. In addition, SA-derived proteins rplU was significantly increased in the vancomycin-treated group relative to the SA-only group (Supplementary Fig. 2E).

**Fig. 3:**
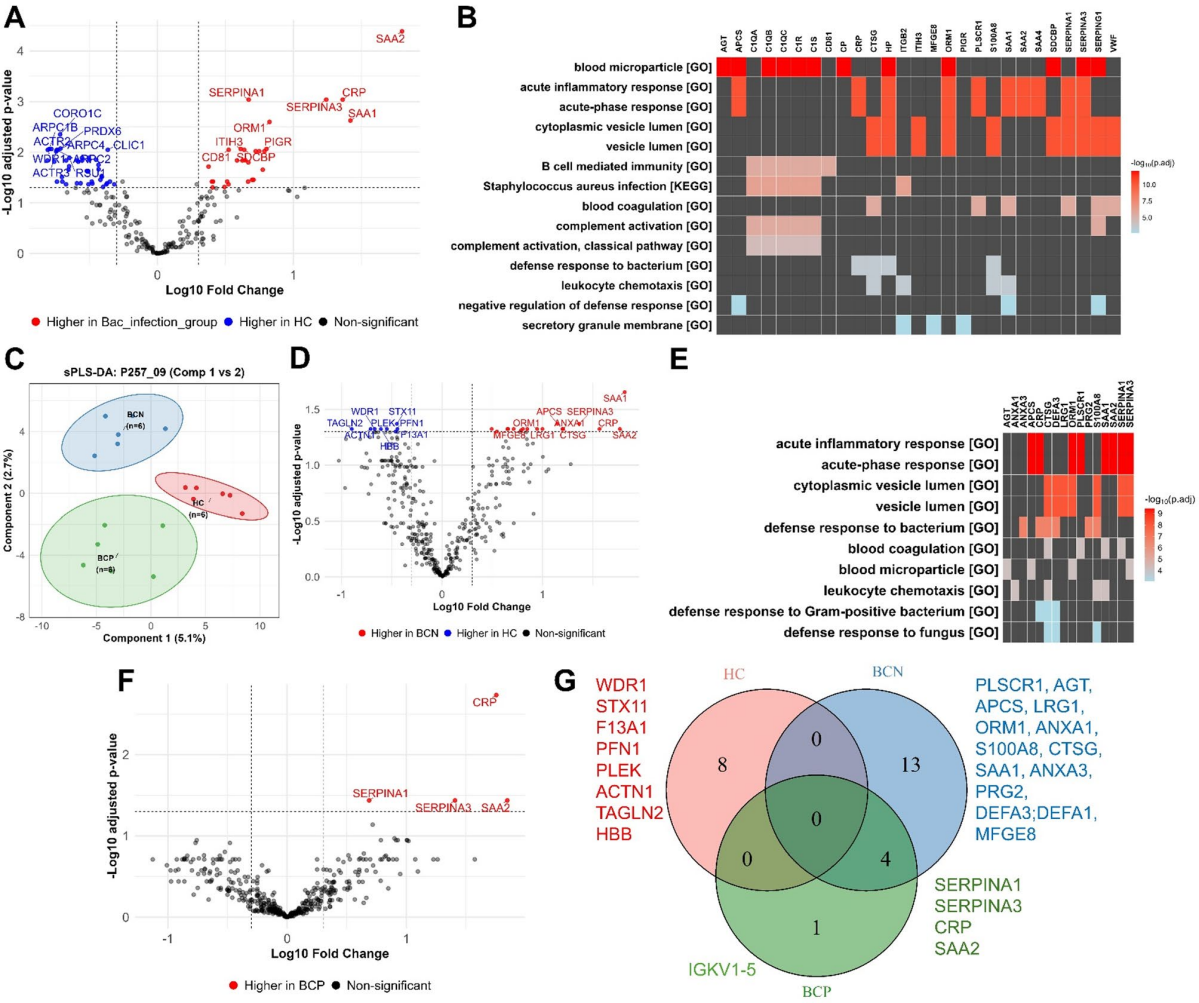
Serum EV proteomes distinguish HC, BCN, and BCP groups. Volcano plots show differential protein expression between (**A**) Bac_infection_group vs. HC, (**D**) BCN vs. HC, and (**F**) BCP vs. HC, with significantly upregulated proteins in red and downregulated proteins in blue; significance was determined using Welch’s t-test with Benjamini–Hochberg (BH) correction based on adjusted p-value and log10 fold change thresholds. Pathway enrichment analysis of significantly upregulated proteins in (**B**) Bac_infection_group vs. HC and (**E**) BCN vs. HC reveals enrichment in blood microparticle, *Staphylococcus aureus* infection, and immune response pathways, assessed using Fisher’s exact test with BH correction, with the color scale representing −log10(adjusted p-value). (**C**) sPLS-DA demonstrates clear separation of EV proteomes among HC, BCN, and BCP samples (*n* = 6 per group), and (**G**) a Venn diagram illustrates the overlap of significantly altered proteins across the three groups.

Since EV–Miltenyi bead flow cytometry and EV proteomics yielded reproducible results in SA–spiked plasma, we next sought to assess whether similar patterns might extend to sepsis patients, including the potential presence of bacterial ribosomal proteins in EVs. Prior to proteomic analysis, EV particle size and number were comparable across HC, BCN, and BCP groups (Supplementary Fig. 3 A, B). Following data preprocessing, including quality control, intensity normalization, and reproducibility-based filtering across biological replicates, proteins were consistently identified across samples from HC (316 ± 74, *n* = 6), BCN (233 ± 31, *n* = 6), and BCP (228 ± 79, *n* = 6). To assess global proteomic changes associated with infection, we compared the combined infection group (BCN + BCP, *n* = 12) to HC (*n* = 6). Differential analysis was performed using Welch’s t-test with Benjamini–Hochberg correction, applying thresholds of adjusted *p* < 0.05 and fold change > 2. This analysis identified 57 proteins enriched in the infection group and 70 enriched in HC (Fig. 3A). Enrichment analysis of infection-associated proteins highlighted pathways related to blood microparticles, positive regulation of exocytosis, *Staphylococcus aureus* infection, secretory granule membrane, innate immune activation, complement activation, and chemotaxis (Fig. 3B).

Notably, several canonical extracellular vesicle–associated and immune-related proteins were more abundant in the infection group, including CD81 and SDCBP (syntenin), together with proteins involved in complement and acute-phase responses such as C1R, C1QA–C1QC, C1S, CP, CRP, APCS, SERPINA1, SERPINA3, SERPING1, ORM1, and HP. Additional proteins linked to immune activation and host defense, including ITGB2, S100A8, CTSG, PIGR, IGHA2, VWF, ITIH3, MFGE8, and members of the serum amyloid A family (SAA1, SAA2, SAA4), were also more abundant, consistent with a coordinated host response to bacterial infection.

sPLS-DA analysis indicated trends toward separation between the HC group and the infection groups (Fig. 3C). A volcano plot further highlighted proteins differentially abundant between BCN and HC, with multiple immune and acute-phase proteins more abundant in the BCN group, including PLSCR1, SERPINA1/3, AGT, CRP, APCS, LRG1, ORM1, ANXA1/3, S100A8, CTSG, SAA1/2, PRG2, DEFA3/DEFA1, and MFGE8 (adjusted *p* < 0.05 and fold change > 2; Fig. 3D). Pathway enrichment analysis of BCN-associated proteins revealed involvement in acute-phase responses, defense responses to Gram-positive bacteria, bacterial and fungal responses, blood coagulation, and vesicle-related pathways (Fig. 3E). Similarly, SERPINA1, SERPINA3, CRP, and SAA2 were more abundant in the BCP group compared to HC (adjusted *p* < 0.05 and fold change > 2; Fig. 3F). A Venn diagram showed that SERPINA1, SERPINA3, CRP, and SAA2 were shared proteins with higher abundance in both BCN and BCP relative to HC (Fig. 3G). Together, these findings suggest that EV proteomes from sepsis patients, regardless of blood culture status, are associated with proteins linked to vesicle-mediated transport, innate immune activation, and host defense pathways compared to HC.

## Discussion

This study presents a multi-tiered investigation into how EVs reflect host responses to bacterial infection and antibiotic treatment, combining an in vitro SA infection model with EV profiling from patients with culture-positive and culture-negative sepsis. Using EV flow cytometry, bead-based isolation, and high-resolution label-free proteomics, we demonstrate that the EV proteome dynamically changes in response to bacterial challenge and is modulated by specific antibiotic treatments. Notably, we identified distinct proteomic signatures in culture-positive and culture-negative sepsis patients that share common immune activation pathways while also exhibiting stratifying features based on blood culture status. Our in vitro data show that SA infection leads to a significant increase in CD45⁺ host-derived EVs carrying bacterial antigens (Fig. 1E), along with increased detection of the SA ribosomal protein RplU in the SA group compared to MOCK (Fig. 2D). In addition, although we observed a significant increase in CMO⁺ PanEV⁻ anti-SA⁺ bMV–BacB complexes in the SA group compared to the MOCK group (Fig. 1F), proteomic readouts from this fraction were limited, restricting further characterization and interpretation. Together, these findings suggest that SA infection promotes the release of host-derived EVs carrying bacterial components, reflecting host–pathogen interactions.

In our SA model, vancomycin and piperacillin-tazobactam, which target the bacterial cell wall and induce membrane stress, showed a tendency toward increased incorporation of bacterial proteins into host EVs in flow cytometry assays, whereas moxifloxacin showed no such effect. This suggests a possible role for cell wall disruption in vesicle-associated protein release, consistent with reports that bactericidal antibiotics, including β-lactams and glycopeptides, can stimulate bMV release as part of a stress response^34^. However, these effects were not clearly reflected at the proteomic level, where no significant differences between antibiotic-treated and SA-only groups were detected after multiple testing correction. Further investigation is required to determine whether EVs and bMVs undergo processing by immune cells prior to analysis, potentially influencing their detectable protein content.

Previous studies using direct bacterial cultures have shown that antibiotic exposure increases vesicle production and cargo release, with β-lactams and other bactericidal agents promoting bMV formation and associated stress responses^34–39^. Although *Staphylococcus aureus* lacks an outer membrane, similar stress-responsive vesicle dynamics have been reported in gram-positive bacteria^34–39^. Consistent with Kim et al.^37^, we detected incorporation of SA-derived ribosomal proteins such as RplU into host EVs, with a tendency toward increased levels following vancomycin treatment (Supplemental Fig. 2E). However, unlike Kim et al.^37^, who used pure bacterial cultures and directly isolated bMVs, our plasma-based approach did not reveal bacterial resistance enzymes and showed no significant proteomic differences between antibiotic-treated and SA-only groups after multiple testing correction. We used the DSM 20,231 strain, a type strain from clonal complex 8 that carries multiple methicillin resistance genes, which may also contribute to differences in vesicle production and protein content compared to other strains. This discrepancy likely reflects the dominance of host proteins in plasma, which can obscure low-abundance bacterial signals, as well as limited bMV protein yield and the lower sensitivity of bulk proteomics compared to particle-based or flow cytometry approaches. Further investigation is required to determine whether EVs and bMVs undergo processing by immune cells prior to analysis, potentially influencing their detectable protein content.

In line with growing evidence that EVs reflect immune dynamics in systemic infection, our study demonstrates that the bacterial infection group, including BCP and BCN sepsis patients, exhibits robust changes in the EV proteome compared to HC. Notably, we observed substantial enrichment of vesicular proteins, innate immune mediators, complement components, and acute-phase proteins in serum EVs, consistent with prior findings summarized in Chang Tian et al.^40^ and other studies.

Notably, several proteins associated with inflammation, immune cell recruitment, and opsonization were elevated in the bacterial infection group compared to HC, including CRP, SAA1, SAA2, SAA4, SERPINA1, SERPINA3, APCS, ANXA1, CD177, ORM1, and AGT. Complement-related proteins such as C1QA, C1QB, C1QC, C1R, C1S, and SERPING1 were also enriched, together with coagulation and vascular injury markers including VWF and ITIH3. Additional proteins linked to vesicle biology and immune activation, including CD81, SDCBP, PLSCR1, HP, CTSG, MFGE8, ITGB2, S100A8, PIGR, and IGHA2, were more abundant in the bacterial infection group relative to HC (Fig. 3). When examined by subgroup, SERPINA1, SERPINA3, CRP, and SAA2 were consistently elevated in both BCP and BCN patients, while the BCN group showed a broader set of enriched proteins, including PLSCR1, AGT, APCS, LRG1, ORM1, ANXA1, ANXA3, S100A8, CTSG, SAA1, PRG2, DEFA1 and DEFA3, and MFGE8. These protein patterns are consistent with EV-associated enrichment of immune and inflammatory mediators during sepsis and support the concept that EVs reflect systemic host responses to infection, although functional roles cannot be inferred from this dataset al.one. CRP, a known common sepsis marker^41,]^^42^ was also detected in our EV proteome, consistent with findings by Yan Xu et al.^42^. However, we did not detect Serine palmitoyltransferase 3 (SPTLC3), possibly due to differences in EV isolation methods, as their study used ultracentrifugation with density gradients, while we employed antibody bead–based capture.

Chanhee Park et al.^43^ and Murao et al.^13^ both identified key EV proteins linked to sepsis, several of which overlap with our findings. These include CRP, SERPINA1, SERPINA3, SERPING1, ORM1, SAA1, SAA2, SAA4, complement components (C1QA, C1QB, C1QC, C1R, and C1S), and ITIH3, all of which were enriched in the bacterial infection group (BCP and BCN) in the sepsis serum EV proteome of this study. SERPINA1 and SERPINA3 are inhibitors of neutrophil serine proteases and have been implicated in regulating inflammation during sepsis^43,44^. In addition, the presence of complement-related proteins such as C1QA, C1QB, C1QC, C1R, C1S, and SERPING1 is consistent with involvement of the classical complement pathway, which has been associated with proinflammatory and prothrombotic responses in sepsis^43,45–47^. These complement components are also linked to pathogen recognition, endothelial activation, immune cell modulation, and regulation of T-cell responses, although functional activity within EVs cannot be determined from the current dataset.

In addition to previously reported proteins, our dataset includes SDCBP (syntenin-1), PLSCR1, CP, HP, AGT, PIGR, IGHA2, APCS, VWF, ITGB2, S100A8, CTSG, CD81, and MFGE8, which may reflect additional or less-characterized components of the host response captured by our antibody bead-based EV isolation method. Notably, SDCBP was more abundant in the bacterial infection group compared to HC (Fig. 3). This observation is consistent with our flow cytometry analysis using EVs isolated by Miltenyi beads (Fig. 1), as well as with our previous publication^48^, in which western blot analysis showed an increasing trend of SDCBP expression in sepsis patients relative to healthy controls. However, despite these changes in protein composition, EV particle numbers remained unchanged in both our previous study and the current dataset (Fig. 1). Together, these findings suggest that sepsis is associated with alterations in EV molecular composition rather than overall vesicle abundance.

Our findings support that PLSCR1, which is overexpressed in sepsis patients compared to HC, plays a key role in protecting lung epithelial cells from SA α-toxin–induced cell death by mediating IFNα-driven resistance mechanisms^49^. Ceruloplasmin (CP), a classic acute-phase protein, has recently been implicated in sepsis-induced cardiotoxicity through its involvement in cuproptosis, a newly identified form of regulated cell death^50^. Haptoglobin (HP) is another well-established acute-phase protein that mitigates hemoglobin-driven oxidative stress^51^. Angiotensinogen (AGT) shows a variable but inducible response to inflammatory stimuli, suggesting context-dependent regulation during acute stress^52^. MFGE8, previously reported to be downregulated in septic patients and protective against oxidative stress and ferroptosis, was found to be elevated in EVs from the BCN and BCP groups in our study; however, the functional significance of EV-associated MFGE8 in sepsis remains to be elucidated^53^.

In conclusion, our study demonstrates that EV proteomes in sepsis are enriched for proteins associated with innate immunity, complement activation, and acute-phase responses, and that bacterial infection is accompanied by alterations in EV composition, including the incorporation of bacterial components. Although differences between culture-positive and culture-negative sepsis were observed, these findings should be interpreted with caution. Owing to the limited sample size and clinical heterogeneity, the study was not powered to identify robust or clinically actionable biomarkers capable of reliably discriminating between these subgroups. Nonetheless, the data provide an initial framework and nominate candidate EV-associated protein signatures that may inform future investigations. Validation in larger, well-characterized cohorts will be required to determine their reproducibility and potential clinical utility for sepsis stratification.

## Supplementary Information

Below is the link to the electronic supplementary material.

Supplementary Material 1

Supplementary Material 2

Supplementary Material 3

## Data Availability

The mass spectrometric raw files as well as the MaxQuant output files have been deposited to the ProteomeXchange Consortium via the PRIDE partner repository and can be accessed using the identifier PXD067303 (reviewer account username: [reviewer\_pxd067303@ebi.ac.uk], password: J7mDqVjcl6XX).[https://proteomecentral.proteomexchange.org/cgi/GetDataset? ID=PXD067303].

## Code availability

The R scripts used for nanoparticle tracking analysis (NTA), extracellular vesicle (EV) flow cytometry, and proteomics data analysis, together with the imputed and normalized ProteinGroups files generated by MaxQuant and Perseus, have been uploaded to GitHub. 10.5281/zenodo.17961832.
